# Anatomical variability of the lingual artery: a comprehensive narrative review with clinical and surgical applications

**DOI:** 10.1007/s00276-026-03846-6

**Published:** 2026-04-13

**Authors:** Viviana Dincă, Rodica Narcisa Calotă, Sorin Hostiuc, Răzvan Costin Tudose, Ivan Varga, Mugurel Constantin Rusu

**Affiliations:** 1 Division of Anatomy, Department 1, Faculty of Dentistry, “Carol Davila” University of Medicine and Pharmacy, 8 Eroilor Sanitari Blvd, 050474 Bucharest, Romania; 2 Institute of Histology and Embryology, Faculty of Medicine, Comenius University, 81372 Bratislava, Slovakia

**Keywords:** Lingual artery, External carotid artery, Linguofacial trunk, Thyrolingual trunk, Hypoglossal nerve, Tongue vasculature, Anatomical variations

## Abstract

**Background:**

The lingual artery (LA) provides primary blood supply to the tongue and floor of the mouth. Precise anatomical knowledge is essential for head and neck surgery and neurointervention to prevent haemorrhage, lingual necrosis, or non-target embolisation.

**Objectives:**

This comprehensive narrative review synthesises current evidence on LA anatomy, variations, and clinical significance whilst critically appraising methodological limitations across the literature.

**Methods:**

A literature search of PubMed/MEDLINE, Scopus, Web of Science, and Google Scholar was conducted from database inception to 15 May 2025. Studies addressing LA anatomy, morphometry, variants, and clinical applications were qualitatively assessed for methodological quality.

**Results:**

The LA typically originates from the external carotid artery at the greater horn of the hyoid bone and courses deep to the hyoglossus muscle. Common arterial trunks represent the most prevalent variants: linguofacial trunk (16–25%), thyrolingual trunk (0.3–3.3%), and thyrolinguofacial trunk (~ 1%). The hypoglossal nerve serves as a reliable surgical landmark, with the LA located inferior to or contacting the nerve in approximately 85% of cases. Dynamic topographic studies demonstrate that tongue extension and surgical retraction significantly alter vessel position, narrowing the midline safety corridor. CTA data suggest tongue extension reduces LA depth beneath the lingual surface by approximately 4 mm (27.9 ± 3.2 mm to 24.0 ± 2.7 mm at foramen cecum level) and decreases bilateral LA distance by approximately 6 mm (20.1 ± 3.1 mm to 13.9 ± 3.2 mm). Critical analysis revealed substantial inconsistencies in prevalence rates for cervical triangles, internal numerical discrepancies in foundational studies, absence of systematic bilateral assessment, and potential non-independence across sequential publications.

**Conclusions:**

Mastery of LA anatomy is indispensable for safe surgical practice. However, clinicians should interpret prevalence data cautiously given methodological limitations, small sample sizes, and potential sample overlap. Future studies with rigorous bilateral assessment and diverse populations are warranted.

**Supplementary Information:**

The online version contains supplementary material available at 10.1007/s00276-026-03846-6.

## Introduction

The external carotid artery (ECA) originates as a terminal branch of the common carotid artery (CCA), usually at the level of the superior border of the thyroid cartilage, and provides the main vascular supply to the extracranial structures of the head and neck. The ECA typically gives rise to eight principal branches: the anterior branches – superior thyroid artery (STA), lingual artery (LA), and facial artery (FA); the medial branch – ascending pharyngeal artery (APA); the posterior branches – occipital artery (OA) and posterior auricular artery (PAA); and the terminal branches – maxillary artery (MA) and superficial temporal artery (STempA) [11, 54]. The branching sequence and morphological configuration of these vessels exhibit significant anatomical variability [11].

**Fig. 1:**
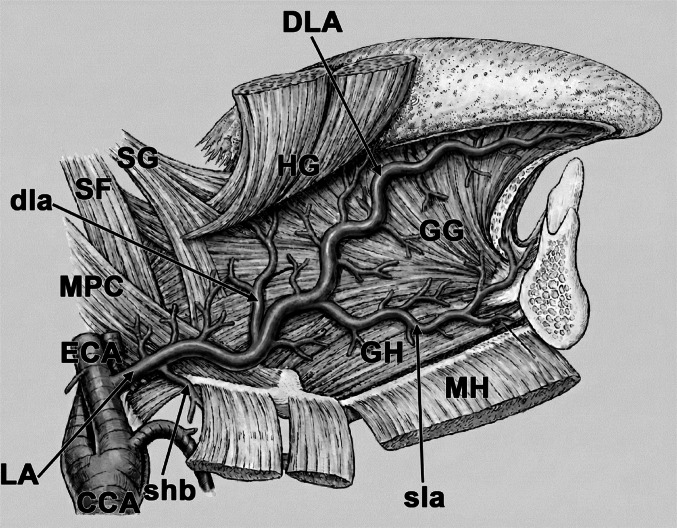
The right lingual artery. Arteries: CCA – common carotid artery; ECA – external carotid artery; LA – lingual artery; DLA – deep lingual artery; dla – dorsal lingual artery (branches); sla – sublingual artery; shb – suprahyoid branch. Muscles: SG – styloglossus; SF– stylopharyngeus; HG – hyoglossus; GG – genioglossus; GH – geniohyoid; MH – mylohyoid; MPC – middle pharyngeal constrictor. Original illustration; anatomical arrangement based on classical depictions

The LA is a significant branch of the ECA, providing the primary blood supply to the tongue and the structures of the floor of the oral cavity (Fig. 1). Precision in identifying its location is paramount in head and neck surgery and neurointervention to prevent complications such as severe haemorrhage, lingual necrosis, or non-target embolisation.

## Methods

### Review design

This article is a comprehensive narrative review of the LA intended to summarise typical anatomy, morphometry, and clinically relevant variants, and to contextualise these findings for contemporary head and neck surgery (e.g., TORS/TOLM, neck dissection) and radiologic practice. Given the heterogeneity of anatomical study designs and reporting standards in this field, we emphasised transparent reporting of search and selection procedures and a critical appraisal of methodological limitations rather than attempting quantitative pooling.

### Literature search strategy

Electronic searches were performed in PubMed/MEDLINE, Scopus, Web of Science, and Google Scholar from database inception to 15 May 2025. Search terms combined free-text keywords and (where applicable) MeSH terms related to the LA and its variants (e.g., “lingual artery”, “linguofacial trunk”, “thyrolingual trunk”, “thyrolinguofacial trunk”, “carotid bifurcation”, “hyoglossus”, “hypoglossal nerve”, “sublingual artery”, “deep lingual artery”, “dorsal lingual artery”, and terms describing tongue surgery and floor-of-mouth haemorrhage), using Boolean operators (AND/OR). Reference lists of eligible full-text articles and key narrative sources were hand-searched to identify additional relevant publications.

To provide screening transparency, the electronic search retrieved 847 records (PubMed: 312; Scopus: 289; Web of Science: 198; Google Scholar: 48). After removal of 234 duplicates, 613 unique records were screened by title/abstract, with 412 excluded (non-human: 89; conference abstracts: 67; insufficient anatomical detail: 156; duplicate publications: 23; non-relevant topics: 77). Full texts were assessed for 201 articles; 106 were excluded (insufficient methodology: 48; unavailable full text: 31; redundant information: 27). Ninety-five publications were included in the final synthesis. A representative PubMed search string is provided in Supplementary Material S1.

### Eligibility criteria

We included human studies (cadaveric dissection, imaging-based studies such as CTA/MRA/angiography, and intraoperative series) reporting on LA anatomy, morphometry, embryology, variants, or clinical/surgical applications. Eligible publication types included original research, case reports/series, and review articles or textbook chapters when they provided substantive anatomical synthesis or unique contextual information. We included articles published in English, French, or German, as well as studies with available English translations. We excluded non-human studies, conference abstracts without full-text availability, duplicate publications without additional primary data, and articles lacking sufficient anatomical detail regarding the LA or its branches.

### Study selection and data extraction

Titles/abstracts were screened for relevance, followed by full-text assessment. For each included study, we extracted: LA origin and course; segmental classifications; morphometric measurements; branching patterns and terminal distribution; prevalence of variants (e.g., common trunks, aberrant origins, atypical trajectories); relationships to adjacent structures (e.g., hypoglossal nerve, hyoid bone, hyoglossus muscle); and reported clinical or surgical implications. Where available, bilateral data and the unit of analysis (patients vs. sides) were recorded, and discrepancies between studies were noted for discussion.

### Terminology and measurement standardisation

For morphometric clarity, the following definitions are applied throughout this review: “calibre” refers to the internal luminal diameter of a vessel; “length” denotes the course distance measured along the vessel axis; and “distance” indicates a straight-line measurement between two anatomical landmarks. Measurements are reported in millimetres (mm); values originally presented in centimetres were converted to mm for consistency.

### Critical appraisal and synthesis

Because included studies varied substantially in design, population, specimen preparation, and measurement methods, we synthesised evidence narratively rather than performing meta-analysis. Findings are organised by anatomical region and clinical relevance, with comparative presentation of major classification systems (e.g., course typologies, segmental models, angiosome concepts). Methodological quality was appraised qualitatively, focusing on sample size, bilaterality of reporting, preservation/fixation effects, demographic characteristics, imaging modality parameters, and internal consistency of reported values. Conflicting results were explicitly highlighted and interpreted in light of likely sources of heterogeneity.

### Ethical considerations

As this study is a review of previously published literature, no ethical approval was required. All data presented are derived from published sources and are appropriately cited.

## Typical anatomy of the lingual artery

The ECA and its branches are functionally analysed as a collection of primary branches with distinct embryologic origins rather than a rigid tree [44]. The LA belongs to the "Oral System" of the ECA framework, alongside the superior thyroid and facial arteries [44].

The LA typically originates from the anterior surface of the ECA at the level of the greater horn (GH) of the hyoid bone. The tip of the greater horn (THB) serves as a constant and reliable surgical landmark for locating the origin [46]. [47] confirmed the hyoid bone's utility as a surgical reference point, noting that the LA is located superior to the hyoid in 89.58% of cases [47]. Modern neurosurgical and angiographic reviews categorise it into four distinct segments [57].

Comprehensive mapping of the carotid sides provides precise morphometric benchmarks for the LA and its surrounding structures. The LA demonstrates a mean internal diameter at its origin of 2.1 ± 0.5 mm and arises, on average, 1.5 ± 5.5 mm inferior to the tip of the greater horn of the hyoid bone and 11.5 ± 6.0 mm posterior to it [16, 46]. The separation between the origins of the lingual and facial arteries is highly variable, with maximum reported values of 14.56 mm on the right side and 11.76 mm on the left [47].

Regarding its relationship to the carotid bifurcation (CB), the LA is located at a mean distance of 12.3 ± 7.3 mm from this landmark [16]. Notably, side-to-side differences are statistically significant, with the LA typically positioned more inferiorly on the left relative to the CB (p = 0.008) [47].

The relationship between the LA and the hypoglossal nerve (HN) is clinically essential for surgical planning. At its origin, the LA is most commonly located inferior to the HN, with a mapped mean separation of 6.9 ± 3.4 mm [16]. Topographic series reports the LA as inferior to the HN in 72.9% of cases, in direct contact with the nerve in 12.5%, and superior to the HN in 14.6% [47].

### Segmental characteristics of the lingual artery

Standard surgical and angiographic models divide the LA course into segments (Table 1) based on its relationship to the hyoglossus muscle and its terminal ascent [57, 77]. [79] refined this for flap reconstruction, identifying four primary segments: the original segment, the hyoglossus segment, and the ascending and horizontal segments of the deep LA [79]. Deep lingual artery–based propeller and axial mucosal flaps have also been described for intraoral reconstruction [19].

**Table 1:** Comparison of segmental classifications of the lingual artery. ECA: external carotid artery

Segment	3-Segment model (Surgical)	4-Segment model (Angiographic)	Anatomical landmarks & features	Citations
I	First segment (retrohyoid)	First segment (retrohyoid)	Original segment (ECA to hyoglossus); forms the "first loop."	[57, 77, 79]
II	Second segment (deep to hyoglossus)	Second segment (deep to hyoglossus)	Hyoglossus segment; runs horizontally deep to the muscle	[57, 77, 79]
III	Third segment (terminal)	Third segment (sublingual)	Ascending segment of the deep lingual artery, anterior to the hyoglossus	[15, 57, 79]
IV	(Included in segment III)	Fourth segment (deep lingual)	Horizontal segment near the tongue tip; forms the "second loop."	[57, 79]

The first segment, also known as the retrohyoid segment, extends from the LA's origin at the ECA to the posterior border of the hyoglossus muscle. This segment typically forms a superiorly convex loop above the greater horn of the hyoid bone and has a mean length of 24.5 mm [77]. Topographic studies demonstrate that the retrohyoid segment lies inferior to the digastric tendon in 97.9% of cases [47], though [32] reported a lower prevalence of 67% for this specific relationship [32]. The second segment courses deep to the hyoglossus muscle and superficial to the middle pharyngeal constrictor, with a mean length of 14.8 mm [77]. The third segment, termed the sublingual segment, travels forward between the genioglossus and mylohyoid muscles [57, 79]. Finally, the fourth segment, also known as the deep lingual or terminal segment, is situated between the genioglossus and the inferior longitudinal muscles and continues toward the tongue tip [57, 79].

### Vascular territories (angiosomes)

The concept of "angiosomes" provides a three-dimensional arterial cartography of the head and neck [38]. The LA is classified as one of the 13 primary angiosomes and is defined as a submerged territory confined to deep tissues (muscles, glands, and specialised organs) without any direct cutaneous representation [38].

Selective injections have defined three primary mucosal territories supplied by branches of the LA. The dorsal LA branches supply the root of the tongue, while the sublingual artery provides blood supply to the ventral surface. The deep LA, in turn, supplies the dorsal aspect of the tongue [50].

The intrinsic blood supply of the tongue follows a distinct architectural pattern, with terminal branches travelling parallel to the muscle fibres (longitudinal, transverse, and vertical) [38].

### Topographical triangles and morphometry

Beyond the classic carotid triangle, several lesser-known cervical triangles (Table 2) can also provide proper surgical access [80, 85]. The level of the CB is highly variable, spanning from 40 mm below to 25 mm above the hyoid bone [17]. On average, the CB lies 13.5 ± 7.5 mm inferior to the tip of the greater horn of the hyoid bone and 17.0 ± 7.0 mm posterior to it [46]. By vertebral level, the CB is most frequently located at C3 (27.21%), C3/C4 (26.19%), and C4 (25.51%) [51].

**Table 2:** Boundaries, significance, and prevalence of neck triangles for lingual artery (LA) identification. HN: hypoglossal nerve; GH: greater horn

Triangle	Boundaries (Ant / Post / Sup / Inf)	Contents (LA related)	Surgical significance	Reported prevalence	Citations
Beclard's	Ant: post. border of the hyoglossus Post: Post. belly of the digastric Inf: GH of the hyoid bone	LA (1st segment), HN	Reliable for proximal ligation and HN identification	91.42% [77] 82.35% [85]	[29, 42, 43, 53, 85]
Pirogoff's	Ant: post. border of mylohyoid Sup: HN Inf: Intermediate tendon of digastric	LA (deep to hyoglossus floor)	Access to the LA in the submandibular region	51.43% [77] 82.35% [85] 58.2% [32]	[29, 42, 80]
Lesser's	Ant/Post: bellies of the digastric muscle Sup: HN	LA (deep to hyoglossus)	Identification of the LA in the submandibular space	65.71% [77] 82.35% [85]	[43, 80, 85]
Farabeuf's	Post: internal jugular vein Inf: common facial vein (or trunk) Sup: HN	CB, ECA, LA Origin	Landmark for high-level vascular procedures	Highly constant	[42, 43, 80]

Intraoperative findings recently reported further indicate that, within Beclard’s and Farabeuf’s triangles, the LA is consistently encountered inferior to the HN, with a mean separation of 5.9 mm [15].

However, the study by [85] examined the prevalence and morphometry of the Beclard's, Lesser's, and Pirogoff's triangles in only 17 formaldehyde-fixed cadavers (34 sides), with a mean age of 78 years and a notable gender imbalance of 5 males and 12 females [85]. The authors reported that Beclard's triangle was present in 82.4% of sides (28/34). In comparison, both Lesser's and Pirogoff's triangles were identified in 88.2% (30/34), with absence attributed to the HN remaining inferior to the digastric muscle. However, a significant internal inconsistency exists within the manuscript: the authors state that Beclard's triangle contained both the HN and LA "when it was present (24 of 24 sides)", despite previously reporting the triangle was present on 28 sides [85], leaving thus four sides unaccounted for. The prevalence of Pirogoff's triangle (88%) in their study [85] is substantially at odds with the findings of [32], who identified the triangle in only 58.2% of 91 dissections from 54 American cadavers [32], a discrepancy the authors acknowledge but do not explain. Furthermore, the relationship of the HN to the digastric muscle demonstrates considerable population-based variability: Tubbs et al. found the nerve inferior to the digastric in only 11.8% of cases [85], whereas [43] reported it inferior in 100% of their French population [43]. Homze et al. found it superior in 58% of cases [32]. These inconsistencies, combined with the study's elderly population, fixation-related tissue alterations, and limited statistical power due to the small sample size and unbalanced gender distribution, suggest that these "forgotten triangles" may not be universally reliable surgical landmarks across populations.

Several concerns arise from comparing the publications of [85] and [42]. The prevalence figures for Beclard's, Lesser's, and Pirogoff's triangles in [42] are not independent primary data; they are reproduced from [85] and therefore do not constitute external validation [42, 85]. More broadly, sequential publications from the same research groups may create the appearance of independent confirmation when the same underlying data and citations are being re-used; readers should therefore consider the potential for non-independence and sample overlap when interpreting cumulative prevalence estimates. Both publications acknowledge the substantial discrepancy between [32], who identified Pirogoff's triangle in only 58.2% of 91 dissections, and the 88.2% prevalence reported by Tubbs et al. [32, 85], but neither paper adequately explains this nearly 30% difference despite similar formalin-fixed cadaver methodology. Furthermore, the internal inconsistency in the original Tubbs et al. paper (Beclard's triangle reported as present on 28 sides yet its contents described in only "24 of 24 sides") remains unaddressed in the Kikuta review, thereby perpetuating an unexplained numerical discrepancy in the literature. These limitations argue for cautious interpretation of these cervical triangles as universally reliable surgical landmarks across populations.

### Seki’s type of course of the lingual artery

**Fig. 2:**
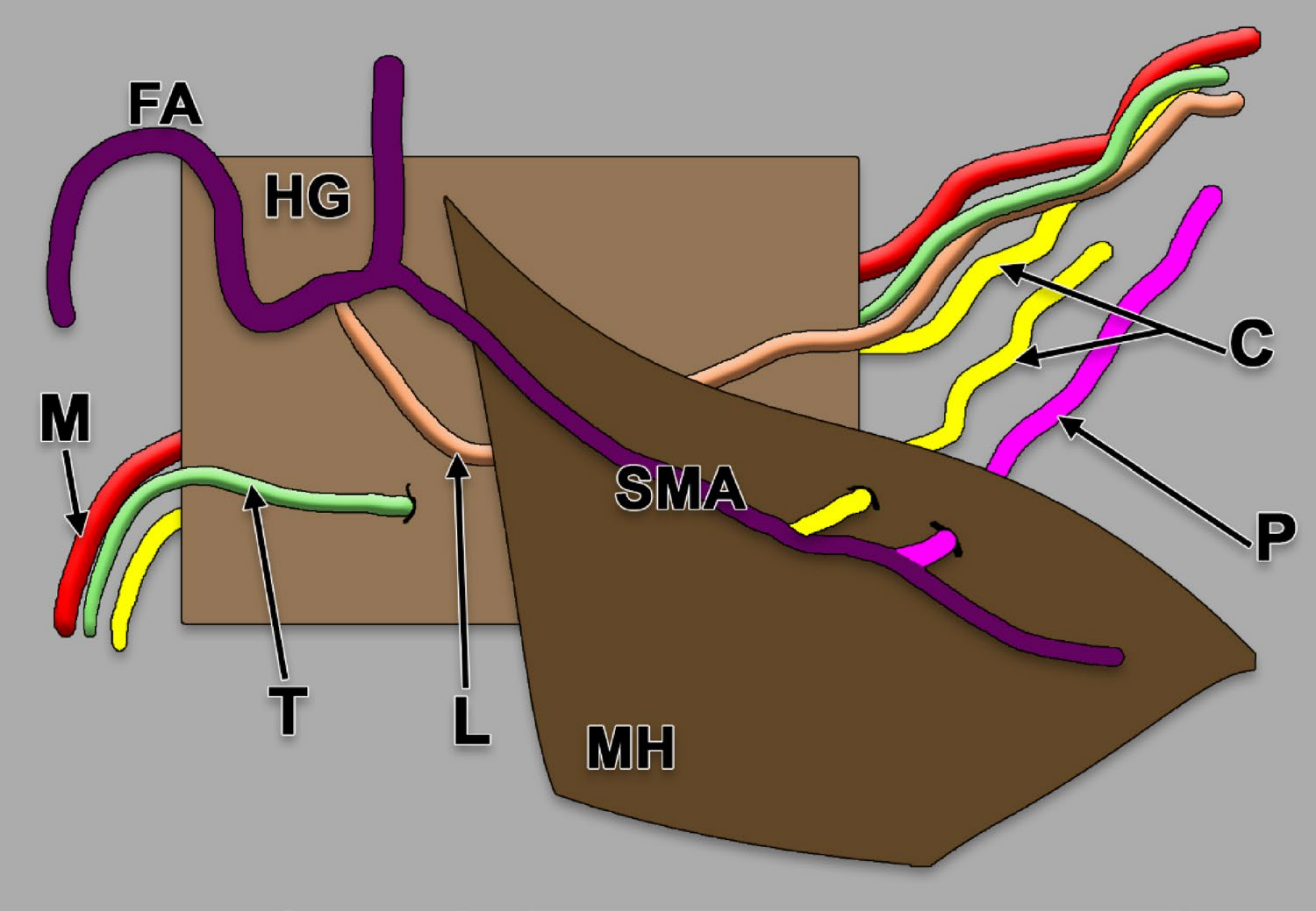
Diagram of Seki’s types (M – medial, L – lateral, T – transferring, P – perforating, and C – combined M and P types) of the course of the lingual artery. Right side, lateral view. FA – facial artery; HG: hyoglossus; MH: mylohyoideus

Seki et al. (72) systematically classified the courses of the human LA based on its relationship with the hyoglossus and mylohyoid muscles, using 111 body sides from 63 Japanese cadavers [78]. Five types were identified (Fig. 2**, **Table 3): Type M, the normal course running wholly medial to the hyoglossus, was observed in 104 sides (93.7%); Type L, coursing wholly lateral to the hyoglossus and originating from the facial artery just proximal to the submental artery, was found in 2 sides (1.8%); Type T, transferring from lateral to medial by penetrating the hyoglossus, also occurred in 2 sides (1.8%); Type P, where the variant LA arises from the distal submental artery and pierces the mylohyoid to reach the sublingual region, was present in 2 sides (1.8%); and Type C, representing the ipsilateral coincidence of both Type M and Type P patterns, was observed in a single side (0.9%). Collectively, variant types (L, T, P, and C) accounted for 6.3% of cases. Notably, the presence of a remnant LA, arising from the ECA in the normal position but terminating as the dorsal LA without continuing to the deep LA, was observed exclusively in Types L and P, indicating these represent "true" anatomical variants where the body of the tongue receives its blood supply from an alternative source, whereas Type T likely represents a partial modification of the normal course related to hyoglossus morphology rather than a fundamentally different vascular pattern [78].

**Table 3:** Classification of lingual artery courses according to Seki et al. (2017), based on the positional relationship with the hyoglossus and mylohyoid muscles. Data derived from gross anatomical dissection of 111 body sides (63 Japanese cadavers). Types L, T, P, and C represent variant courses (combined prevalence 6.3%), with the presence of a remnant lingual artery distinguishing true vascular variants (Types L and P) from morphological modifications (Type T). Abbreviations: LA, lingual artery; ECA, external carotid artery; FA, facial artery

Type	Description	Course	Origin of variant LA	Frequency (n = 111)	Prevalence	Remnant LA present
M	Medially coursing to the hyoglossus	Wholly medial to the hyoglossus; it enters deep at the posterior border and reappears at the anterior border	ECA (normal)	104 sides	93.7%	No
L	Laterally coursing to the hyoglossus	Wholly lateral to the hyoglossus from the posterior to the anterior border	FA (just proximal to the submental artery origin)	2 sides	1.8%	Yes
T	Transferring from lateral to medial	Lateral at the posterior part of the hyoglossus, then penetrates the muscle to course medially	ECA	2 sides	1.8%	No
P	Penetrating the mylohyoid	Pierces through the mylohyoid to enter the sublingual region	Distal portion of the submental artery	2 sides	1.8%	Yes
C	Coincidence of M and P	Both normal (type M) and variant (type P) LAs are present ipsilaterally	Submental artery (for variant component)	1 side	0.9%	No

### Dynamic topography and surgical mapping

In patients with obstructive sleep apnea–hypopnea syndrome (OSAHS) and oropharyngeal tumours, the LA position appears highly responsive to tongue posture and intraoperative manipulation [37, 93]. Moving the tongue from a resting to a fully extended position significantly reduces the depth of the LA beneath the lingual surface (p < 0.01) [93]. At the foramen cecum level, Wu et al. (2015) reported a reduction in depth from 27.9 ± 3.2 mm to 24.0 ± 2.7 mm (approximately 4 mm) and a reduction in the distance between bilateral lingual arteries from 20.1 ± 3.1 mm to 13.9 ± 3.2 mm (approximately 6 mm), narrowing the midline safety corridor [93].

To improve safety in robotic surgery, Hou et al. (2012) defined the “V zone” corridor—an anatomic working space located above the “Big Dipper” configuration of the LA and the hypoglossal-lingual neurovascular bundle (HLNVB) [35]. The V zone is subdivided into a V prozone (anterior to the circumvallate papillae) and a V postzone (the tongue-base corridor). In the resting position, the V postzone has a reported safe width of 31.42 ± 3.82 mm [35] (Table 4).

**Table 4:** Comparison of LA topography in tongue resting vs. extended/retracted positions. LA = lingual artery; GH = greater horn (of the hyoid); BOT = base of tongue; V_post = posterior “V” zone/postzone; raphe = midline raphe

Landmark / Metric	Resting position	Extended / Retracted position	Clinical change / Implication	Key citations
Morphological pattern	“Big Dipper” (“Plough”) configuration	“Check-mark” (√) configuration	Significantly superior/anterior (upward/forward) shift	[36, 37]
LA origin to GH	Higher relative position	Lower relative position	Mean decline of 9.27 mm	[37]
Depth beneath the surface	≈10 mm at BOT	Reduced depth (more superficial)	Vessels move closer to the surgical field	[18, 93]
Distance to the midline	≈19 mm at BOT	Lateral shift	Retraction pulls bundles laterally	[18, 64]
Midline safety corridor	V postzone (V_post): ~ 31.4 mm (mean safe width)	Narrowed / more superficial	Increased risk near the midline raphe	[35, 93]

High-resolution 3D contrast-enhanced CT (3D-CECT) mapping by [64] further refined the anatomy of the base of the tongue (BOT). As the LA courses posteriorly through the BOT, it follows an “outward and downward” path. At the level of the foramen cecum, the artery lies 19.2 ± 3.1 mm from the midline raphe, at a depth of 13.5 ± 2.8 mm. More posteriorly, at the vallecula level, the distance from the midline increases to 24.8 ± 4.2 mm [64].

With surgical retraction using a Feyh-Kastenbauer (FK) retractor, the HLNVB can shift substantially. [18] reported that the bundle is typically located approximately 19 mm lateral to the midline and 10 mm deep at the BOT; however, retraction displaces it further laterally and more superficially, thereby reducing the safe midline corridor [18].

### Branches and terminal course of the lingual artery

The LA gives rise to several branches along its course before terminating as the deep LA. The suprahyoid branch originates from the first segment and courses along the superior border of the hyoid bone. The dorsal lingual branches arise from the second segment deep to the hyoglossus muscle and supply the root of the tongue [50]. [79] noted that the root of the tongue receives supply from two to three branches of the LA, supplemented by contributions from the ascending palatine and tonsillar arteries [79]. The detailed territory of the dorsal lingual branches consists of the dorsum and posterior tongue, soft palate, and adjacent mucosa. These arteries are critical in base‑of‑tongue surgery and help explain patterns of haemorrhage and safe zones.

The sublingual artery arises at the anterior margin of the hyoglossus muscle. It provides blood supply to the sublingual gland, the floor of the mouth, and the ventral surface of the tongue (Table 5) [50]. The sublingual artery runs anteriorly on the lingual aspect of the mandible together with the sublingual vein, beneath/around the mylohyoid and within the sublingual space [82]. In vivo MR angiography and CT show it most often branching in the molar region and coursing forward toward the canine–premolar region, paralleling dentition [82]. Its mean arterial caliber (“major axis”) is about 24 mm, and vessels are larger and more frequently visualized in males [82]. In approximately 11% to 15% of cases, the sublingual artery originates from the FA as a common sublingual-submental trunk [3, 77]. Mapping of the arterial supply to the floor of the mouth classifies three patterns: Type I, in which the sublingual artery is dominant (71%), Type II, in which the submental artery is dominant (7%), and Type III, in which both arteries contribute equally (22%) [41]. The sublingual artery was found to be small, missing, or insignificant in 53% of cases; in such cases, a large perforating branch from the submental artery is present [5].

**Table 5:** Key topographic relations of the sublingual artery

Structure	Relation to the sublingual artery	Relevance	Citation
Lingual cortex of the mandible	The artery runs closely along the inner surface	Implant and genioplasty risk	[82]
Mylohyoid muscle	The artery/branches may pass deep or pierce near the canine	Explains variable bleeding points	
Sublingual vein	Often accompanies the artery, hard to separate on CT/MRA	Bleeding may appear “venous”	

The sublingual artery serves as the primary vascular pedicle for the sublingual gland flap [90], and its termination frequently forms the lingual frenal artery at the midline [79]. The lingual frenal artery is a small terminal branch of the sublingual/lingual arterial system that courses close to the lingual frenulum to supply the anterior ventral tongue and adjacent floor of mouth mucosa, and although often not described separately in classic angiographic work, it belongs to the rich arterial network to the floor of the mouth and lingual gingiva that can cause significant hemorrhage if transected during midline incisions, frenectomy or implant surgery in the anterior mandible [5].

The sublingual and submental arteries form a clinically important anastomotic network across the mylohyoid muscle, reinforcing blood supply to the anterior mandible, floor of mouth, tongue and sublingual gland and explaining the robust vascularity of submental and floor-of-mouth flaps. Cadaveric work shows that in almost half of mylohyoid muscles, branches of the submental or sublingual arteries traverse the muscle via mylohyoid boutonnières, where they pass between the submandibular and sublingual spaces to supply intraoral structures [95]. These trans-mylohyoid branches represent potential sites of anastomosis between the facial-system submental artery and the lingual-system sublingual artery, creating a collateral arcade that can maintain perfusion even if one source is partially compromised [82, 95]. Imaging studies confirm that sublingual and submental arteries run in close parallel along the lingual aspect of the mandible and beneath the mylohyoid and anterior belly of digastric, providing opportunities for segmental interconnections and explaining why injury to either vessel during dental implant placement or flap elevation can lead to brisk hemorrhage and extensive sublingual–submental hematoma [82]. For reconstructive surgeons, this anastomotic pattern underlies the reliability of the submental artery flap for intraoral reconstruction and mandates careful identification and ligation of branches piercing the mylohyoid when raising myocutaneous flaps that include this muscle [95].

The superior laryngeal artery is typically a branch of the STA; however, it arises from the LA in approximately 7% of cases [16]. [23] documented computed tomography angiography (CTA) evidence of a unilateral superior laryngeal artery originating from a suprahyoid coil of the LA [73]. The LA origin of the superior laryngeal artery has also been documented in various dissection studies [22, 40, 59, 61, 72, 88]. Additionally, a rare unilateral variation has been reported in which an accessory branch to the infrahyoid muscles left from the root of the LA [10].

The deep LA (ranine artery) represents the terminal segment of the LA [30] and is typically highly tortuous, angiographically described as a "second loop". It supplies the body of the tongue through an average of approximately 25 arterial branches [79] and constitutes the primary arterial supply to the dorsal tongue [50]. Histopathologic mapping demonstrates a systematic change in vessel trajectory: as the artery courses from the tip toward the base of the tongue, it travels progressively deeper and more laterally, with mean depth increasing from 4.2 mm at the tip to 14.6 mm at the base [55]. While the dorsal branches course posteriorly and superiorly to the dorsum and root of tongue, the deep LA runs anteriorly toward the tip of the tongue [30].

Regarding anastomoses and septal relationships, the internal structures of the tongue are generally separated by the lingual septum. Lead oxide injection studies revealed that the tongue possesses an almost avascular midline, effectively rendering each half a vascular end organ [38]. Although a robust dorsal submucous arterial network exists [79], actual vascular crossover at the midline raphe is minimal [38]. Robust extrinsic anastomoses occur around the base of the tongue via the FA, as well as in the floor of the mouth through FA branches [63].

The tongue is overall highly vascular, yet several anatomical and imaging studies support the concept of a relative midline watershed, where major arterial branches are sparse, with the dominant supply arising from paired territories of each LA on either side of the tongue rather than crossing extensively at the midline [18, 93]. During base of tongue and midline glossectomy procedures, CTA has shown that the bilateral Las course closer together and more superficially when the tongue is fully extended, but still remain lateral to the true midline at key reference points such as the foramen cecum region, reinforcing the notion that the central raphe is comparatively less vascular than adjacent paramedian zones [93]. Cadaveric work mapping the HN-LA neurovascular bundle similarly demonstrates that the dorsal branches of the LA are encountered more laterally and become more superficial with retraction, thereby allowing surgeons to define a midline “safe zone” in which the risk of direct arterial injury is lower, even though small anastomoses and capillary networks persist [18]. Clinically, this architecture underpins both the feasibility of midline approaches for sleep surgery and oncologic resections and the parallel practice of prophylactic LA ligation in neck dissection, which can significantly reduce the risk of catastrophic haemorrhage without causing global tongue ischemia, since contralateral and collateral channels maintain adequate perfusion despite interruption of one main trunk [15, 24] (Table 6).

**Table 6:** Tongue vascularity and surgical landmarks. LA: lingual artery

Topic	Key finding related to midline vascularity	Citations
Vascular resilience after ligation	Unilateral LA ligation does not cause gross tongue atrophy, implying robust collateral supply, mainly lateral but sufficient across midline	[24]
Artery position vs tongue posture	With full tongue extension, lingual arteries move closer to each other and the surface, yet remain lateral to midline landmarks	[93]
Safe zones in BOT surgery	Dorsal lingual branches become more superficial and are not encountered in a defined posterior midline zone, supporting a relative avascular corridor	[18]
Neck dissection ligation strategy	The LA usually lies deep to or just inferior to the hypoglossal nerve, enabling elective ligation to protect against transoral hemorrhage	[15]

## Variational anatomy of the lingual artery

### Common trunks of the lingual artery

The carotid arterial system demonstrates considerable anatomical variability, with numerous documented variations in the branching patterns of the ECA. Among these variants, common arterial trunks formed by the fusion of adjacent branches are clinically significant anomalies that can affect surgical and interventional procedures in the head and neck region. According to Lippert and Pabst (1985), the linguofacial trunk (LFT) may be encountered in 18% of cases, the thyrolingual trunk (TLT) with ECA origin in 2%, and the thyrolinguofacial trunk (TLFT) in < 1% [48].

#### The linguofacial trunk

The LFT global prevalence is estimated between 16 and 25% [23, 47]. The TLT is rare, with reported incidence rates ranging from 0.3% to 3.3% [62, 94]. Ectopic origin from the CCA occurs in < 0.1–0.25% of cases [48, 84]. High CBs are often associated with these common trunks [20, 81].

#### The thyrolingual trunk

The TLT may arise from either the ECA or, more rarely, from the CCA (Table 7). When originating from the CCA, the trunk is classified as an ectopic variant due to its low position of origin relative to the carotid bifurcation. Calotă et al. (2025) identified two cases of TLT in their study of 170 carotid axes: one from the CCA and another from the ECA. In the case of CCA origin, the TLT originated from below the GH horn, with a trunk length of 5.1 mm [11]. The trunk divided into the LA and STA at 5 mm lateral to the superior horn of the thyroid cartilage; thus, the ascending pharyngeal artery becomes the first branch of the ECA [11]. In the second case, the TLT originated from the ECA immediately superolateral to the hyoid tubercle [11].

**Table 7:** Summary of TLT prevalence in anatomical studies. CB = carotid bifurcation; N/A = not available. TLT = thyrolingual trunk; CCA = common carotid artery; ECA = external carotid artery; CB = carotid bifurcation; N/A = not available. *Contralateral ECA morphology was presented but without detailed branch sequencing

Author (year), Ref	Sample size	TLT cases	Prevalence	Origin(s)	Bilateral assessment
Quain (1844), [68]	302	1	0.3%	N/A	No
Livini (1903), [49]	200	3	1.5%	N/A	No
Poynter (1922), [67]	200	1	0.5%	N/A	No
Aaron and Chawaf (1967), [1]	187	11	5.9%	N/A	No
Poisel and Golth (1974), [66]	156	5	3.2%	N/A	No
Lemaire et al. (2001), [45]	Case report	1	-	CCA	No
Vázquez et al. (2009), [88]	330	2	0.6%	ECA (1), CCA (1)	No
Natsis et al. (2011), [60]	100	3	3.0%	ECA (1), CB (1), CCA (1)	No
Iwai et al. (2012), [39]	265	1	0.4%	CCA	No
Nochikattil et al. (2017), [62]	Case report	1	-	CCA	No
Herrera-Núñez et al. (2020), [31]	152	16	10.5%	N/A	No
Cobiella et al. (2021a), [16]	193	2	1.0%	ECA (1), CCA (1)	No
Tsakotos et al. (2024), [84]	400	1	0.25%	CCA	Partial*
Rusu et al. (2024), [75]	Case series	1	-	ECA	No
Calotă et al. (2025), [11]	170	2	1.18%	CCA (1), ECA (1)	Yes

A consistent limitation across prior TLT reports (Table 7) is the absence of a systematic bilateral assessment. Neither Vázquez et al. [60], nor [16] documented contralateral vascular morphology [16, 60, 88]. Case reports by [39] and [62] similarly omitted detailed bilateral ECA branching sequences [39, 62, 84, 31], despite reporting the highest TLT prevalence (10.5%), did not document ipsilateral branch sequencing [31, 74]described a right ECA-origin TLT associated with hyoid-laryngeal overlap but likewise did not detail bilateral branching patterns [75].

#### The thyrolinguofacial trunk (TLFT)

The prevalence of the TLFT is ~ 1%. It can arise from the CB [6, 20] or the CCA [81]. A rare bilateral TLFT has been reported [6] (Table 8).

**Table 8:** Chronological comparison of reported thyrolinguofacial trunk cases from the carotid bifurcation or common carotid artery

Author (Year)	Method	Origin site	Citations
Iwai et al. (2012)	3D-CTA	1.6 mm below CB	[39]
Cvetko (2013)	Cadaver	At High CB	[20]
Suresh and Murugan (2017)	Cadaver	From CCA (High CB)	[81]
Baxla et al. (2018)	Cadaver	Anterior ECA (Bilateral)	[6]

#### The thyrolinguolaryngeal trunk

A thyro-linguo-laryngeal trunk was recently found unilaterally by dissection [9]. It arose from the ECA and trifurcated into the STA, LA and superior laryngeal artery [9]. It may be regarded as a subvariant of a TLT, in which the superior laryngeal artery does not originate distally from the STA.

#### The laryngolingual trunk

Rusu et al. (2024) documented by CTA a superior laryngeal artery leaving a suprahyoid coil of the LA and continuing over the GH horn to enter the larynx through the thyrohyoid membrane; the STA originated from the left ECA immediately above the CB [73]. Soon after, [59] found by dissection and reported the common origin of the superior laryngeal artery and the LA, in the form of a trunk, from the ECA, in coexistence with a STA origin from the CB [59]. They termed „laryngolingual” the common trunk.

### Rare courses and hyoid associations

Classically, the LA leaves the ECA postero-superiorly to the hyoid tubercle, and in 93% of cases enters deeply to the hyoglossus muscle at its posterior margin (type M, medially coursing) [78]. In 1.8% of cases, it initially runs laterally and superficially along the posterior aspect of the hyoglossus, then turns medially into the muscle (type T, transferring from lateral to medial) [78]. In another 1.8% of cases, it runs completely laterally to the hyoglossus (type L, laterally coursing) [78]. When these topographical types were originally established, the relationship between the LA and the greater hyoid horn was either overlooked or the artery was invariably found superior to the greater horn [74, 78].

Vascular trajectories may be influenced by elongated styloid processes or ossified hyoid segments [74]. The stylohyoid complex may comprise several distinct osseous/ossified elements: the tympanohyal, stylohyal, ceratohyal, and the hypohyal just above the hyoid [21]. Any styloid process longer than 30 mm is radiologically classified as elongated, a finding strongly associated with Eagle (stylohyoid) syndrome, which arises when either a calcified stylohyoid ligament or excessive styloid length irritates adjacent neurovascular structures [25–27]. The clinical presentation depends on the direction of the elongated process: a laterally deviated styloid process can compress the ECA [28].

In a case report featuring bilateral elongated styloid processes (right: 56.3 mm; left: 38.0 mm), atypical LA trajectories associated with ossified hyoid segments were found [74]. On the right, the LA originated from the ECA lateral to the greater horn (GH) of the hyoid, describing a unique inferoposterior coil before continuing anteriorly along the lateral aspect of the greater horn for 12.1 mm; it then looped anteriorly and ascended above the greater horn to pierce the hyoglossus muscle, thereafter continuing with a typical course [74]. This pattern corresponds to Seki’s type T of LA course [78]. This transferring variant initially courses laterally and superficially along the posterior aspect of the hyoglossus before turning medially into the muscle and is documented in only 1.8% of cases [78]. The novel observation was that the initial type T course was not along the hyoglossus itself but along the lateral surface of the greater hyoid horn [74]. On the left, the LA followed a typical course but crossed laterally over an ossified hypohyal, a 17.3 mm ossified fragment situated just superior to the hyoid body [74]. This represents a rare anatomical configuration where the artery's trajectory is constrained to pass superficially over an ossified stylohyoid chain element.

### Sequential patterning of ECA branches

In Calotă et al.'s recent study, 40 different types of sequence of the proximal branches of the ECA were distinguished. The LA with independent origin from the ECA was found in 30/40 of those types, whilst the LA originating from common trunks (TLT, LFT) was found in 10/40 sequencing types [11]. In all types where the LA has an independent origin, it is preceded by at least one other branch (most commonly the STA) [11]. Notably, earlier work by Wolf et al. (1985) reported that the LA represents the most proximal branch in 53.8% of cases [92].

### Axial spin of the CB and ECA’s lateral transposition: impacts on the lingual artery departure

Rotation of the CB ("axial spin") ranges from -45° to 90° [52]. In cases of lateral transposition **(**3.6% to 5.3%**)**, the LA must undertake a modified lateral-to-medial course [76]. Clinical complications include peripheral hypoglossal nerve palsy [87]. "Twisted" CBs can develop progressively over time due to arterial elongation [33, 86].

## Embryological and functional considerations

The third and fourth aortic arches establish the primary vascular architecture of the head and neck. According to Houseman et al. (2000)**,** the first definitive vascular arch in this region is a loop formed between the internal carotid artery (derived from the third aortic arch) and the vertebral artery (derived from the portion of the fourth aortic arch that becomes the subclavian artery) [38]. This paired arcade is linked ventrally by the circle of Willis and dorsally by the basilar system to ensure brain perfusion [38].

The ECA emerges from the common carotid derivative of the third arch. While the carotid system is the primary driver [2, 4, 70, 83], arch stabilisation requires cardiac neural crest cell ensheathment; its absence leads to arterial destabilisation and loss of bilateral symmetry [7, 8, 89].

Mechanical growth forces dictate the ultimate course and shape of the LA during prenatal development [38]. Brain growth stretches vessels from the skull base toward the vertex, resulting in a curved arrangement of the ophthalmic and occipital branches. Concurrently, the lateral and ventral growth of the pharyngeal arches, specifically the mandibular and maxillary processes, mechanically "drags" their supplying vessels forward, explaining the characteristic forward trajectory of the LA and MA toward the oral and nasal cavities. As skeletal maturation proceeds, the increasing vertical height of the cervical spine elongates vessels such as the CCA, while others become entrapped within maturing bony canals [38].

## Clinical significance

### Interventional considerations

Anatomical variants of the LA carry significant clinical implications for interventional and surgical procedures. A LA-origin superior laryngeal artery, occurring in approximately 7% of cases, or sublingual-submental trunks, present in 15%, create particular risks during superselective catheterisation [3, 16]. The collateral circulation of the tongue presents a paradox: robust extrinsic anastomoses and the dorsal submucous network can permit tissue survival following radical surgery, yet these same communications may compromise hemostasis during unilateral vessel ligation [63, 79]. Despite these anastomotic networks, the LA retains an essentially end-organ nature, with limited midline crossover, such that sacrifice of a single LA carries a significant risk of partial tongue necrosis, particularly when collateral supply from the FA is insufficient [38]. From a hemodynamic perspective, loss of the dicrotic notch in the arterial waveform serves as a marker of increased arterial stiffness [56].

### Surgical applications

The LA’s surgical applications span a broad spectrum of head and neck procedures, each demanding precise anatomical knowledge to minimize morbidity. Real-time intraoperative ultrasound guidance has been proposed to aid vascular identification during TORS for obstructive sleep apnea–hypopnea syndrome [14]. Elective ligation of the LA has been advocated prior to transoral robotic surgery (TORS) or transoral laser microsurgery (TOLM), providing prophylactic hemorrhage control [15]. When intraoral control of haemorrhage proves unfeasible, extraoral ligation serves as a critical fallback manoeuvre [47]. In approaching the vessel, the tuberculum of the hyoid bone represents a reliable landmark for locating both the LA and the hypoglossal nerve [46, 47].

In elective neck dissection, the HN is a reliable landmark: in 63.6% of 33 cases, the LA runs directly deep to the nerve; in another 21.2% it lies within 5 mm inferior; only 6.1% are superior [15]. This supports a practical rule: search just deep to, or slightly inferior to, the HN in level II–III dissection fields to locate the LA for ligation [15]. In 25 patients undergoing TORS with unilateral ligation, CT-based measures of muscle density (a surrogate for vascularity) showed no significant difference in overall tongue vascularity between ligated and non-ligated sides, and no gross tongue atrophy or major complications were seen at ≈4-month follow-up [24]. Ligation of LA/FA was not associated with first bite syndrome; parapharyngeal fat manipulation was the key risk factor instead [91]. Therefore, the extraoral ligation of the LA is anatomically feasible and clinically useful, particularly in neck dissection combined with transoral oncologic surgery. The HN is the most practical intraoperative landmark, with the LA usually immediately deep or slightly inferior. Short- to mid-term data suggest that prophylactic ligation does not significantly compromise tongue vascularity or cause major functional complications, though long-term effects remain less clearly studied.

The lingual sulcus release, a transcervical approach employed for tongue cancer resection, requires precise identification of the LA and hypoglossal nerve to minimise surgical morbidity [71]. For base-of-tongue surgery, the three-dimensional LA map developed by [64] has emerged as a highly effective planning tool for TORS procedures [64]. Complementary digital 3D models of the oral tongue and floor of mouth have been created by integrating radiology, anatomical literature, and medical illustration to support surgical planning [53]. By estimating arterial depth and lateralisation at specific landmarks, approximately 13.5 mm deep at the foramen cecum, for instance, surgeons can plan base of tongue reduction or tumour resection with substantially reduced risk of catastrophic haemorrhage.

Adherence to established safe zones is paramount during tongue surgery. Surgeons should respect the V-zone corridor, recognising that tongue retraction narrows the "safe" midline corridor as the HN and lingual neurovascular bundle become more superficial and shift laterally [18, 35, 93].

Specific procedural contexts carry particular vascular risks. At the tongue tip, the deep LA lies in close proximity to both the midline raphe (approximately 2.5 mm) and the dorsal surface (approximately 4.2 mm), substantially increasing the likelihood of vascular injury during distal tongue procedures [55]. During mid-neck surgery, lateral transposition of the ECA or LA, as well as an elongated styloid process, can increase the risk of inadvertent vascular injury [74, 87].

Dental implants, bone grafting, and genioplasty in the canine–premolar region risk lingual cortical perforation with injury to the sublingual (and submental/deep lingual) arteries, which can cause life-threatening floor-of-mouth hematoma and airway obstruction. Non-contrast MR angiography (SSFP with time-SLIP) can preoperatively map sublingual, submental, and deep lingual arteries with good agreement to contrast CT, potentially identifying patients at higher hemorrhage risk before implant placement [82]. Knowledge that sublingual arteries cluster in the canine–premolar zone, while deep lingual branches favor the molar region and submental branches the incisor region, can guide safer implant site selection and drilling angulation [82].

In dental surgery, endosseous implants placed in the anterior mandible can precipitate severe floor-of-mouth haemorrhage; in approximately 10% of patients, vessels in this region exceed 1.5 mm in diameter, thereby amplifying the potential severity of bleeding if injured [41, 58]. A prospective CBCT-based study of 50 edentulous patients found that 88% of planned midline implants touched the lingual canal, yet 12% had profuse bleeding; life-threatening events in the literature are mostly associated with implants > 15 mm and definite lingual cortex perforation [13].

The deep LA runs forward along the ventral tongue and floor of the mouth, close to the lingual cortex of the anterior mandible (Table 9). Imaging and anatomical studies show that branches of the LA (including the deep lingual and sublingual arteries) and the FA (submental artery) are the main blood supply to the anterior mandible [13, 82]. Non-contrast MR angiography demonstrated that the deep lingual, sublingual, and submental arteries run just 1–2 mm from the lingual mandibular cortex, tracking along the lingual side of the incisor, canine, premolar, and molar regions, often contiguous along the arch [82]. When an endosseous implant or drill perforates the lingual cortical plate, it may lacerate any of the three arteries. It can cause a rapidly expanding floor-of-mouth hematoma, airway compromise, and, rarely, death [13, 82]. Because the deep LA (and sister branches) lie so close (≈1–2 mm) to the lingual cortex of the mandible, even small perforations in the anterior mandible can disrupt vessels ≥ 1.5 mm in diameter and precipitate severe haemorrhage [13, 69, 82].

**Table 9:** Comparison of dorsal and deep lingual branches

Branch	Main territory	Clinical relevance	Citations
Dorsal lingual arteries/branches	Dorsum and base of tongue, posterior mucosa	At risk in base-of-tongue resections, TORS	[14, 18]
Deep lingual artery (ranine)	Ventral/anterior 2/3 of tongue	At risk in anterior tongue surgery, lingual flaps	[19, 30]

### Pathological and diagnostic considerations

Vascular malformations of the tongue constitute approximately 7% of all benign tumours in the head and neck region [12]. Lesions can lead to spontaneous oral haemorrhage and macroglossia. MRI is the preferred diagnostic modality to delineate lesion extent relative to the LA [12].

Benign tumours and tumour-like lesions of the tongue are uncommon overall; however, vascular malformations, including venous, lymphatic, and arteriovenous types, constitute a recognised subset that may present with macroglossia, haemorrhage, airway compromise, dysphagia, and speech or masticatory dysfunction [65]. Pediatric series confirm that vascular malformations and hemangiomas feature among surgically treated tongue lesions, with diffuse lesions often manifesting as macroglossia and occasionally precipitating respiratory distress [34].

Cross-sectional imaging, particularly magnetic resonance imaging, is emphasised as crucial for characterising benign tongue lesions and defining their full deep extent [65]. For vascular malformations specifically, MRI permits differentiation between low-flow and high-flow (arteriovenous) malformations based on signal characteristics and the presence or absence of flow voids [65]. In arteriovenous malformations, imaging allows visualisation of serpiginous flow voids and feeding arteries, often supplemented by MR angiography or CT angiography to demonstrate arterial feeders and the nidus [65]. Although detailed metrics relative to the LA are not always systematically tabulated, imaging-based depiction of feeding lingual branches and their proximity to the lesion underpins treatment planning, whether surgical, embolic, or laser-based, to effectively control hemorrhage risk [65].

## Conclusions

The LA exhibits considerable anatomical variability in origin, course, and branching patterns. The tip of the greater horn of the hyoid bone and the hypoglossal nerve remain the most reliable surgical landmarks, with the artery located inferior to or in contact with the nerve in approximately 85% of cases. Common arterial trunks represent the most prevalent origin variants, with the linguofacial trunk occurring in 16–25%, the thyrolingual trunk in 0.3–3.3%, and the thyrolinguofacial trunk in approximately 1% of individuals. Seki's classification provides a useful framework for understanding trajectory variants relative to the hyoglossus muscle [78].

The dynamic topography of the LA during tongue manipulation has significant surgical implications, as extension and retraction narrow the midline safety corridor and bring vessels closer to the mucosal surface. In CTA-based measurements, tongue extension reduced the depth of the LA beneath the lingual surface by approximately 4 mm (27.9 ± 3.2 mm to 24.0 ± 2.7 mm at the foramen cecum) and decreased the distance between bilateral lingual arteries by approximately 6 mm (20.1 ± 3.1 mm to 13.9 ± 3.2 mm), highlighting a measurable loss of midline safety margin during transoral procedures [93]. These values derive from 17 OSAHS patients and require validation in larger surgical series. Accordingly, surgical safe-zone guidance and prophylactic ligation recommendations should be considered preliminary clinical observations rather than definitive guidelines. Prophylactic ligation during neck dissection, combined with transoral surgery, is anatomically feasible without significant compromise of tongue vascularity. The sublingual-submental anastomotic network across the mylohyoid muscle explains both collateral perfusion capacity and haemorrhage risk during anterior mandibular procedures.

Gaps in the literature include inconsistent bilateral assessment, small sample sizes, and methodological limitations in foundational studies of cervical triangles. For TORS/TOLM practitioners, dynamic topography data from Wu et al. (2015) demonstrate that tongue extension reduces the depth of the LA beneath the lingual surface by approximately 4 mm (from 27.9 ± 3.2 mm to 24.0 ± 2.7 mm at the foramen cecum level) and decreases the distance between bilateral lingual arteries by approximately 6 mm (from 20.1 ± 3.1 mm to 13.9 ± 3.2 mm), significantly narrowing the midline safety corridor [93]. These values derive from CTA measurements in 17 OSAHS patients and require validation in larger surgical series. Recommendations regarding prophylactic ligation and surgical safe zones should be considered preliminary clinical observations rather than definitive guidelines. Future research using advanced imaging in diverse populations will further refine understanding of LA variants. Mastery of normal and variant LA anatomy remains indispensable for safe surgical and interventional practice in the head and neck region.

## Supplementary Information

Supplementary Material 1

## Data Availability

The datasets used and analyzed during the current study are available from the corresponding author upon reasonable request.

## Abbreviations

APA: Ascending Pharyngeal Artery
BOT: Base of Tongue
CB: Carotid Bifurcation
CCA: Common Carotid Artery
CEA: Carotid Endarterectomy
CECT: Contrast-Enhanced Computed Tomography
ECA: External Carotid Artery
FA: Facial Artery
FK: Feyh-Kastenbauer (retractor)
GH: Greater Horn (of the hyoid bone)
HLNVB: Hypoglossal/Lingual Artery Neurovascular Bundle
HN: Hypoglossal Nerve
ICA: Internal Carotid Artery
LA: Lingual Artery
LFT: Linguofacial Trunk
LSR: Lingual Sulcus Release
MRI: Magnetic Resonance Imaging
OA: Occipital Artery
OPT: Occipitopharyngeal Trunk
OSAHS: Obstructive Sleep Apnea-Hypopnea Syndrome
SA: Sublingual Artery
SGF: Sublingual Gland Flap
SLA: Superior Laryngeal Artery
SMA: Submental Artery
STA: Superior Thyroid Artery
THB: Tip of the Greater Horn (of the hyoid bone)
TLFT: Thyrolinguofacial Trunk
TLT: Thyrolingual Trunk
TOLM: Transoral Laser Microsurgery
TORS: Transoral Robotic Surgery
