# Supplementary material for: Anatomical variability of the lingual artery: a comprehensive narrative review with clinical and surgical applications
Source: Surg Radiol Anat. 2026 Apr 13;48(1):118. doi: 10.1007/s00276-026-03846-6 (PMC13076371; doi:10.1007/s00276-026-03846-6)
Supplement: Supplementary file 1 — Supplementary Material 1 [file 276_2026_3846_MOESM1_ESM.docx]

# Supplementary Material S1

Representative PubMed/MEDLINE search strategy for the lingual artery narrative review.

Database: PubMed/MEDLINE

Search executed: 15 May 2025

Results at time of search (as recorded by authors): 312

Fields: Title/Abstract and MeSH Terms (where available)

## Search string

(("lingual artery"[Title/Abstract] OR "arteria lingualis"[Title/Abstract] OR "lingual artery"[MeSH Terms]) AND (anatomy[Title/Abstract] OR anatomical[Title/Abstract] OR variation*[Title/Abstract] OR variant*[Title/Abstract] OR morphometr*[Title/Abstract] OR "linguofacial trunk"[Title/Abstract] OR "thyrolingual trunk"[Title/Abstract] OR "thyrolinguofacial trunk"[Title/Abstract] OR "sublingual artery"[Title/Abstract] OR "deep lingual artery"[Title/Abstract] OR "dorsal lingual artery"[Title/Abstract] OR "hypoglossal nerve"[Title/Abstract] OR hyoglossus[Title/Abstract] OR "carotid bifurcation"[Title/Abstract] OR TORS[Title/Abstract] OR "transoral robotic"[Title/Abstract] OR "transoral laser"[Title/Abstract] OR TOLM[Title/Abstract] OR haemorrhage[Title/Abstract] OR hemorrhage[Title/Abstract] OR emboli*[Title/Abstract] OR ligation[Title/Abstract]))

Note: This is a representative string intended to illustrate the core query structure. Minor adaptations (synonyms, spelling variants, and field tags) were applied across databases to accommodate platform-specific syntax.
